# Neuroprotective and Angiogenesis Effects of Levetiracetam Following Ischemic Stroke in Rats

**DOI:** 10.3389/fphar.2021.638209

**Published:** 2021-05-14

**Authors:** Xiang Yao, Wenping Yang, Zhendong Ren, Haoran Zhang, Dafa Shi, Yanfei Li, Ziyang Yu, Qiu Guo, Guangwei Yang, Yingjiang Gu, Hairong Zhao, Ke Ren

**Affiliations:** ^1^ Department of Radiology, Xiang’an Hospital of Xiamen University, Xiamen, China; ^2^ Division of Neurology, Department of Geriatrics, Jiangsu Province Hospital, First Affiliated Hospital of Nanjing Medical University, Nanjing, China; ^3^ Hospital (T.C.M) Affiliated to Southwest Medical University, Luzhou, China; ^4^ School of Medicine, Xiamen University, Xiamen, China

**Keywords:** ischemic stroke, levetiracetam, neuroprotection, hypoxia-inducible factor, heat shock protein 70, vascular endothelial growth factor

## Abstract

**Objective:** The present study explored whether levetiracetam (LEV) could protect against experimental brain ischemia and enhance angiogenesis in rats, and investigated the potential mechanisms *in vivo* and *in vitro*.

**Methods:** The middle cerebral artery was occluded for 60 min to induce middle cerebral artery occlusion (MCAO). The Morris water maze was used to measure cognitive ability. The rotation test was used to assess locomotor function. T2-weighted MRI was used to assess infarct volume. The neuronal cells in the cortex area were stained with cresyl purple. The anti-inflammatory effects of LEV on microglia were observed by immunohistochemistry. Enzyme-linked immunosorbent assays (ELISA) were used to measure the production of pro-inflammatory cytokines. Western blotting was used to detect the levels of heat shock protein 70 (HSP70), vascular endothelial growth factor (VEGF), and hypoxia-inducible factor-1α (HIF-1α) in extracts from the ischemic cortex. Flow cytometry was used to observe the effect of LEV on neuronal cell apoptosis.

**Results:** LEV treatment significantly increased the density of the surviving neurons in the cerebral cortex and reduced the infarct size (17.8 ± 3.3% vs. 12.9 ± 1.4%, *p* < 0.01) after MCAO. Concurrently, the time required to reach the platform for LEV-treated rats was shorter than that in the saline group on day 11 after MCAO (*p* < 0.01). LEV treatment prolonged the rotarod retention time on day 14 after MCAO (84.5 ± 6.7 s vs. 59.1 ± 6.2 s on day 14 compared with the saline-treated groups, *p* < 0.01). It also suppressed the activation of microglia and inhibited TNF-α and Il-1β in the ischemic brain (135.6 ± 5.2 pg/ml vs. 255.3 ± 12.5 pg/ml, 18.5 ± 1.3 pg/ml vs. 38.9 ± 2.3 pg/ml on day 14 compared with the saline-treated groups, *p* < 0.01). LEV treatment resulted in a significant increase in HIF-1α, VEGF, and HSP70 levels in extracts from the ischemic cerebral cortex. At the same time, LEV reduced neuronal cell cytotoxicity and apoptosis induced by an ischemic stroke (*p* < 0.01).

**Conclusion:** LEV treatment promoted angiogenesis and functional recovery after cerebral ischemia in rats. These effects seem to be mediated through anti-inflammatory and antiapoptotic activities, as well as inducing the expression of HSP70, VEGF, and HIF-1α.

## Introduction

Ischemic stroke is the second most common cause of death worldwide and the main cause of disability (Grysiewicz et al., 2008; Warner et al., 2019). Although there has been great progress in understanding the mechanism of brain injury after ischemia, the development of new drugs for the treatment of ischemic stroke has not progressed as rapidly. The mechanisms of cell death caused by cerebral ischemic injury are complicated and include excitotoxicity, ion imbalance, oxidative stress, and inflammation (Anrather and Iadecola, 2016; Jayaraj et al., 2019; Yang et al., 2019). Due to the increasing prevalence of ischemic stroke in the general population and the lack of adequate treatment methods, it is imperative to research and develop more effective drugs for this neurodegenerative disease.

Angiogenesis is a key component of poststroke neurovascular remodeling processes in which new capillaries are formed through directed proliferation and migration of endothelial progenitor cells from preexisting blood vessels (Ruan et al., 2015; Dąbrowski et al., 2019). Vascular endothelial growth factor (VEGF) is one of the key pro-angiogenic factors that increase after ischemia in both rodent and human brains. VEGF is regulated by the transcriptional hypoxia-inducible factor-1 (HIF-1), which regulates gene transcription to facilitate adaptation and survival after hypoxia–ischemia (Hong et al., 2019). Heat shock protein 70 (HSP70) in mice has been shown to provide protection from cerebral ischemia in an animal model of stroke, suggesting that there is a correlation between induction of HSP and resistance to damage (Doeppner et al., 2017; Shao et al., 2019).

Levetiracetam (LEV) is often used to treat epilepsy. In cell and animal models of neurodegenerative diseases, LEV has been proven to have neuroprotective properties at the therapeutic level (Stöllberger and Finsterer, 2016; Kapur et al., 2019). LEV also has therapeutic and protective effects on cerebral hemorrhage (Shetty, 2013).

In this study, we investigated whether LEV had a neuroprotective effect on rats with ischemic stroke. In addition, we correlated the possible neuroprotective effects of LEV with the induction of HSP70, VEGF, and HIF-1α expression, and the inhibition of microglia activation.

## Methods

### Animal Model Preparation and LEV Treatment

**FIGURE 1:**
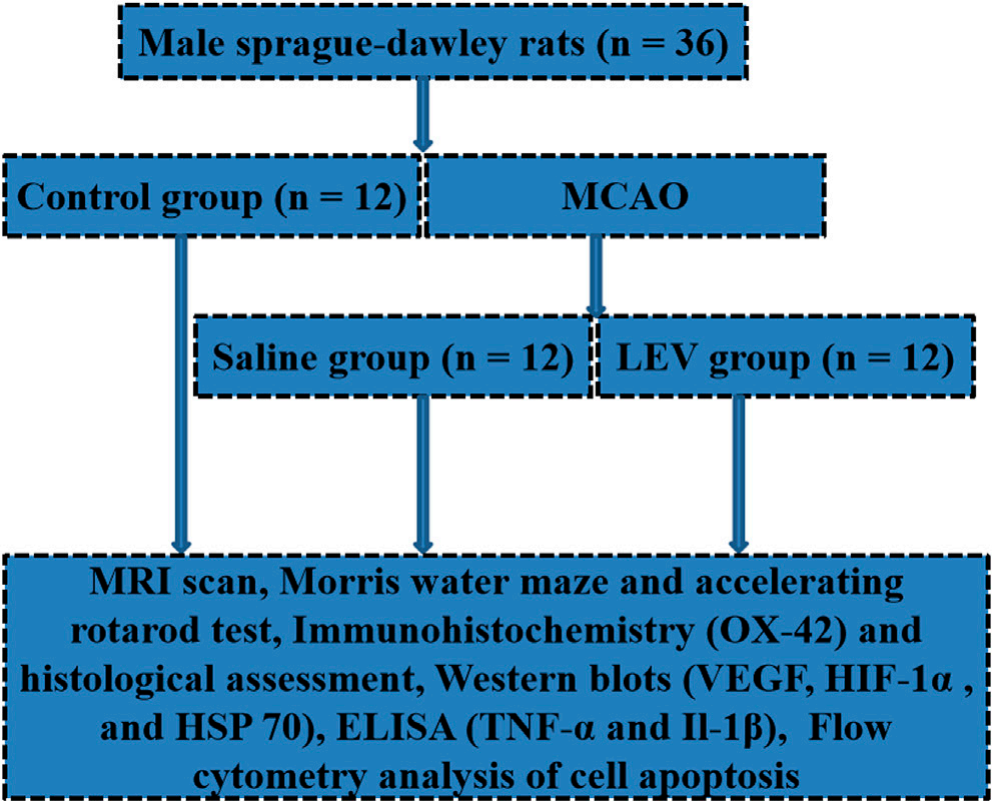
Schematic of the study design, including all regimens.

All studies were approved by the Animal Ethics Committee. Male Sprague–Dawley (SD) rats (200–220 g, Xiamen University Laboratory Animal Center, Xiamen, China) were used and divided randomly into three groups: 1) control group (*n* = 12), 2) saline-treated group (*n* = 12), and 3) LEV-treated group (*n* = 12). A schematic of the study groups including all regimens is presented in Figure 1. The acclimatization time for rats in the study was 2 weeks. SD rats underwent middle cerebral artery occlusion (MCAO) surgery on the right neck under inhalation anesthesia (70% N_2_O and 30% O_2_ containing 1.5% isoflurane) (Tsai et al., 2011; Wang et al., 2011). The right common carotid artery, external carotid artery, and internal carotid artery were exposed through a midline incision. A 4–0 nylon suture with a flame-rounded tip was inserted into the right internal carotid artery through the right common carotid artery, and then into the Willis Circle to block the origin of the right middle cerebral artery. Ischemia was ensured by monitoring the loss of the righting reflex and bilateral pupil dilation during carotid artery occlusion. After 60 min, the suture was removed. During the operation, the temperature of the rats was maintained at 37.0 ± 0.5°C by a heating blanket. The duration of reperfusion was 24 h. In the control group, there was no arterial occlusion, and other operations were the same. After the rats were completely recovered from anesthesia, neurological severity was evaluated immediately. There was no significant difference in the recovery time from anesthesia based on the observations among the groups. Rats without neurological deficits were excluded from the study. LEV (150 mg/kg; A606205; Sangon Biotech, Shanghai, China) was injected immediately at the beginning of the reperfusion phase, 12 h later, and then once daily for up to 14 days (Inaba et al., 2019). The saline group was given an intraperitoneal injection of the same amount of saline.

### MRI and Data Analysis

On the 14th day after MCAO, the rats were anesthetized (1.5% isoflurane) and stationed on a mechanical ventilator. The body core temperature was maintained at 37.0 ± 0.5°C using a heated circulating water pad. The rats were placed in a whole process for MR scanning, and the entire process was monitored. The rats were placed in a stereotactic stent for magnetic resonance imaging (MRI) scanning, and the entire MRI process was monitored. The MRI experiments were performed on a horizontal bore 9.4 T scanner operating on a Bruker Avance platform (Bruker 9.4 T Biospec). T2-weighted imaging was used to evaluate the infarct volume. Three mutually perpendicular images were obtained to localize the infarction site. Then, 13–15 T2-weighted axial slices (1 mm thick) covering the entire damage area (field of view [FOV] = 30 × 30 mm, matrix size = 256 × 256, echo time [TE] = 33 ms, and repetition time [TR] = 3,500 ms) were acquired by a spin-echo pulse sequence to delineate anatomical details and calculate qualitative T2 maps.

### Morris Water Maze and Accelerating Rotarod Test

The Morris water maze task was evaluated as previously described with slight modification (Morris, 1984). The rats were screened before surgery to identify those that could not swim. The trials were conducted during days 8–11 after ischemia. In each trial, the task of all animals was to find a hidden, transparent plastic platform (10 cm diameter) placed 50 cm (150 cm in diameter and 60 cm in depth) from the water maze wall and 1 cm below the water. All animals were randomized every day in the same order. These animals faced the pool wall before being released, and the time required to reach the hidden platform was recorded. During the experiment, the animals were allowed to rest on the platform for 30 s. If the rat failed to reach the platform within 120 s, it would be guided to the platform manually. Each rat received two sessions of treatment every day for four consecutive days.

The motor skills and coordination ability in the MCAO rats were measured with an accelerating rotarod apparatus (Ugo Basile, Italy); the speed was accelerated from 0 to 40 rpm for more than 4 min (Puig et al., 2018). The rats received once-daily training sessions of 3 trials separated by 30-min intervals for 3 consecutive days before MCAO. The longest time each rat stayed on the rod was recorded as the baseline. One, 7, and 14 days after MCAO, rats were tested on the rotarod three times, and the best performance of each rat on that day was recorded.

### Immunohistochemistry and Histological Assessment

On the 14th day after ischemia, the rats were euthanized, and the brains were removed and fixed in 4% buffered paraformaldehyde for 72 h. Samples were then dehydrated, embedded in paraffin, cut into 5 μm sections, and finally stained with cresyl violet. The number of normal neuronal cells in the penumbra cortex was counted under a microscope (Leica, Leica DM2700 P, Germany).

Immunohistochemical staining was performed on the 14th day after ischemia. The sections were microwave-washed for 5 min, washed three times in PBS (pH 7.4), and then incubated with 0.3% hydrogen peroxide in methanol for 10 min and 10% normal goat serum in PBS for 20 min. Then, the sections were incubated with primary antibody (OX-42, 1:1,000, ab1211; Abcam, United Kingdom) dissolved in 2% normal goat serum, 0.3% Triton X-100, and 0.05% NaN3 in PBS at 4°C overnight, and incubated at 37°C for 30 min (Xuan et al., 2012). When the microglial cells were in a resting state, the specific marker OX-42 positive cells were stained very lightly, with small cell bodies, small protrusions, and unclear cell morphology and contours, making it difficult to identify. While the nerve is noxiously stimulated, the microglial cells will undergo changes in morphology and function, which can cause a significant upregulation of the OX-42 on the surface of microglial cells. After washing three times in PBS, the sections were incubated with secondary antibody at 37°C for 30 min in PBS, and then with avidin-biotin-peroxidase solution, and stained with the 3,3′-diaminobenzidine (DAB) kit.

### Western Blots

The cerebral cortex tissue was dissected and sonicated in tissue protein extraction reagent. The lysates were centrifuged at 12,000 rpm for 10 min at 4°C, and the supernatants were extracted for immunoblotting. The protein concentration was determined by the bicinchoninic acid (BCA) method, and the samples were boiled at 95°C for 5 min. Equal amounts of protein were separated by gel electrophoresis on 8–12% sodium dodecyl sulfate-polyacrylamide gels and transferred to polyvinylidene difluoride membranes by wet transfer for 60 min. The nonspecific binding sites were blocked with 5% skimmed milk at room temperature for 1 h. Then the membrane was incubated with the following primary antibodies overnight: VEGF (1:1,000, ab32152; Abcam, United Kingdom), HIF-1α (1:500, NB100–105; Novus Biologicals, Centennial, United States), and HSP70 (1:1,000, ab2787; Abcam, United Kingdom) at 4°C overnight. After washing with PBS, the membranes were incubated with secondary antibody for 1 h at room temperature. An enhanced chemiluminescence system was used to visualize the immunostained protein bands, and the bands were analyzed with ImageJ software. Beta-actin was used as an internal control.

### Measurement of Pro-Inflammatory Cytokines by ELISA

The cerebral cortex samples were sonicated (10 w, 2 × 5 s) in a mixture containing protease inhibitors (20 mg/ml each of pepsin A, aprotinin, phosphoramide, and leupeptide; 0.5 mm PMSF; and 1 mm EGTA) and then centrifuged at 100,000× *g* for 20 min at 4°C. Interleukin-1β (IL-1β) and tumor necrosis factor-α (TNF-α) were measured in the supernatant by ELISA (R&D Systems, United States) on the 14th day after ischemia (Wang et al., 2012).

### Flow Cytometry Analysis of Cell Apoptosis

To induce oxygen-glucose deprivation (OGD), PBS was used to wash the culture medium, and deoxygenated glucose-free DMEM medium was used instead. The cultures were then placed in an incubator chamber containing a mixed gas of 5% CO_2_ and 95% N_2_ at 37°C for 3 h. The neuronal cells in the control group were placed in glucose-containing DMEM medium for the same period of time. After 3 h, the neuronal cells were returned to normal oxygen conditions and reperfused for 24 h. For the LEV-treated neuronal cells, the drug was added to the cells at a final concentration of 1 mmol/L at the beginning of OGD and replenished immediately after reperfusion (Wang et al., 2019). Neuronal cells were seeded in 6-well plates at a density of 1 × 10^5^ cells/well. The treated neuronal cells were stained with the Annexin V-Light 650 Cell Apoptosis Detection Kit (Wanleibio; WLA002b, Shenyang, China). After incubating for 15 min in the dark, the apoptosis rate was measured by flow cytometry (Bechman, MoFlo Astrios EQS, United States).

### Statistical Analysis

All data were analyzed with SPSS17.0 or GraphPad Prism 8 and expressed as the mean ± standard deviation. Statistical analysis of the data was performed using one-way analysis of variance (ANOVA) followed by the least significant or the Student–Newman–Keuls test. *p* < 0.05 was statistically significant.

## Results

### LEV Treatment Reduces Cerebral Infarct Volume and Neuronal Death Induced by Ischemic Stroke in Rats

**FIGURE 2:**
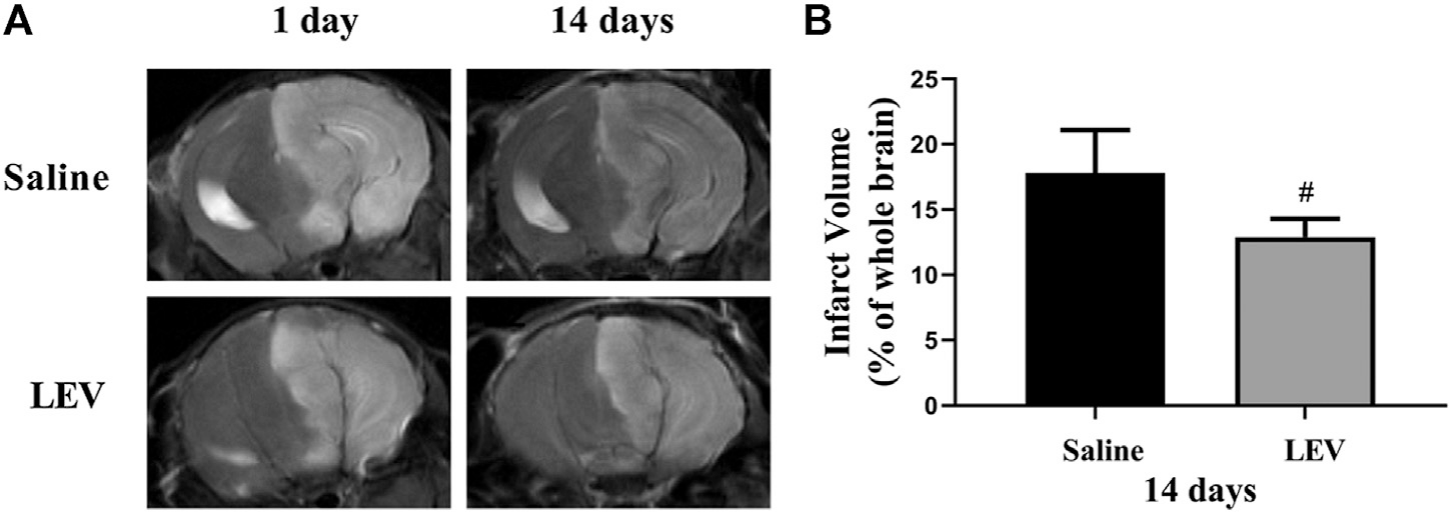
Cerebral infarction with levetiracetam (LEV) treatment reduced cerebral infarct volume in middle cerebral artery occlusion (MCAO) rats. **(A**,**B)**. Brain infarct volume was detected by T2-weighted magnetic resonance imaging (MRI). LEV treatment significantly reduced the cerebral infarct volume of MCAO rats on the 14th day. *N* = 12 per group. ^#^ *p* < 0.01 compared to the saline group.

**FIGURE 3:**
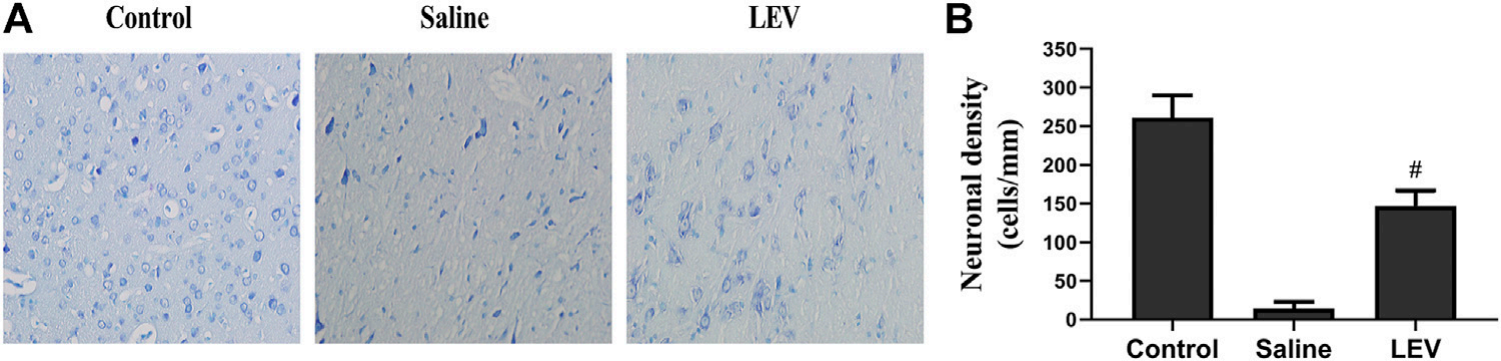
Levetiracetam (LEV) treatment after cerebral infarction reduced neuronal death. **(A)** Cresyl violet staining of the cerebral cortex in the control, saline, and LEV groups (original magnification: ×400). **(B)** Quantification of cresyl violet stained cells in the penumbra of the cerebral cortex 14 days after cerebral ischemia. *N* = 12 per group. ^#^ *p* < 0.01 compared to the saline and control groups.

Fourteen days after ischemic stroke, the T2 images showed that cerebral infarction in the saline group accounted for 18% of the whole brain. After LEV treatment, the cerebral infarction size significantly reduced to 13% (Figures 2A,B). Histological analysis showed that the neuronal density in the ipsilateral cortex area of the saline group was significantly lower than that in the control group (Figure 3B). Cresyl violet staining showed unequivocal signs of neuronal death with pyknotic and shrunken nuclei. Compared to the saline group, the density of the neurons in the cerebral cortex in the LEV group was significantly increased, and the signs of neurons with shrunken nuclei were also improved (Figure 3A).

### LEV Treatment Alleviates Deficits in Spatial Cognitive Function Induced by Ischemic Stroke and Promotes Functional Recovery

**FIGURE 4:**
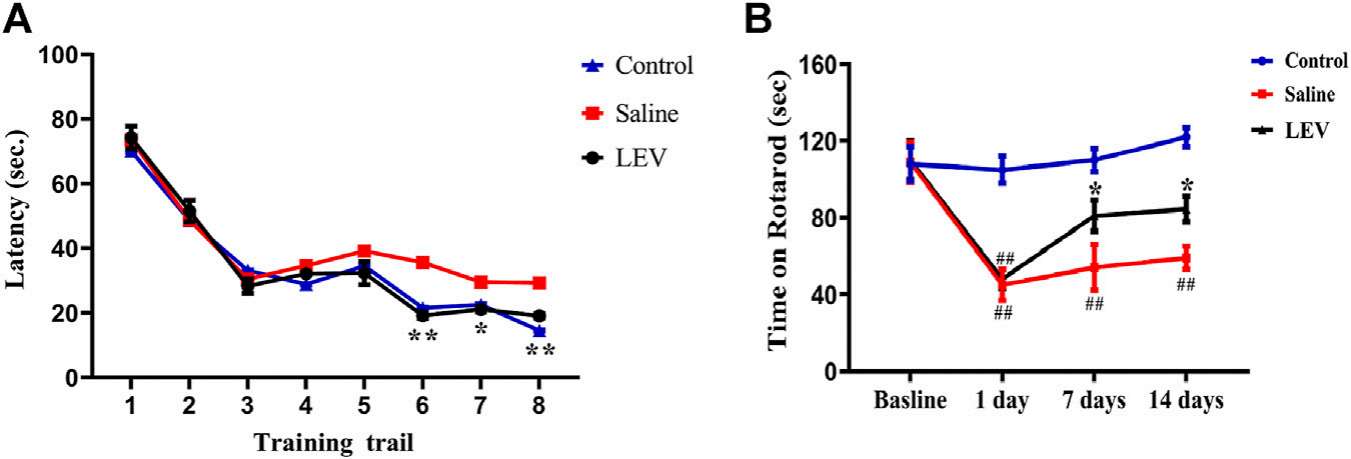
Levetiracetam (LEV) treatment improved neurological function recovery and attenuated behavioral defects after middle cerebral artery occlusion (MCAO)-induced cerebral infarction. The water maze task and rotarod test were used for evaluation. **(A)** LEV significantly shortened the time required for rats to reach the platform in the water maze task. **(B)** LEV markedly increased the retention time of MCAO rats on an accelerating rotarod on days 7 and 14 after ischemia. *N* = 12 per group. *p < 0.05; **p < 0.01 LEV group vs. saline group; ^##^ *p* < 0.01 compared to the control group.

To evaluate the effect of LEV on the surviving neurons and the functional state of the brain, the Morris water maze was used to assess learning and memory in the rats. The rats in the control group reached the hidden platform in a short time during training. In contrast, rats in the saline group showed obvious functional defects. The time required to reach the platform for LEV-treated rats was shorter than that in the saline group but not differ from that of the control group (Figure 4A). On day 14 after MACO, the time the rats were able to stay on the accelerating rotarod was significantly shortened (Figure 4B). LEV treatment after ischemia effectively improved this motor function defect by prolonging the rotarod retention time.

### LEV Exhibits Anti-Inflammatory Effects in the Treatment of Ischemic Brains

**FIGURE 5:**
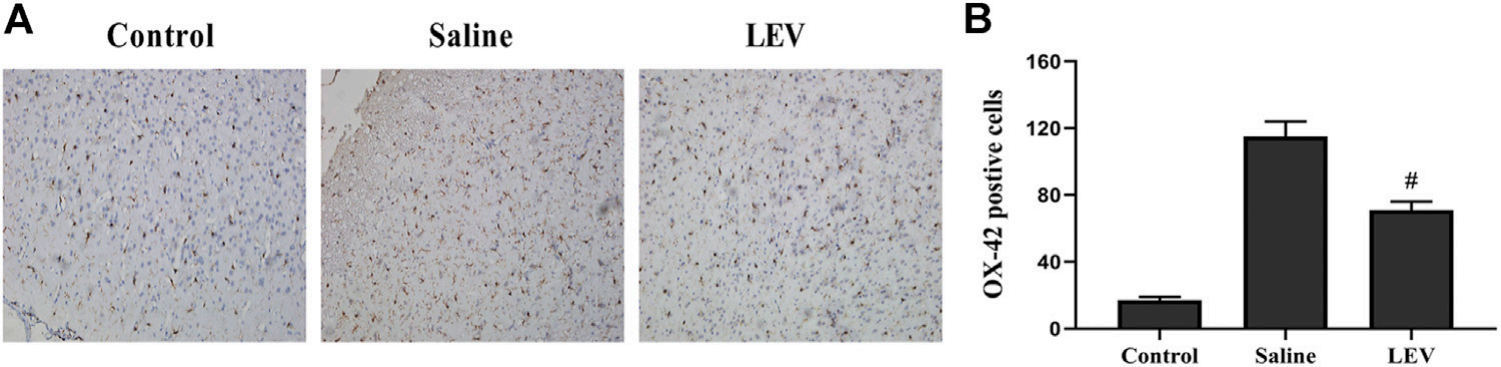
Levetiracetam (LEV) exhibits anti-inflammatory properties in the cerebral cortex of the ischemic brain. **(A)** Photographs of OX-42–immunopositive cells in the cortex after cerebral infarction (original magnification: ×200). **(B)** The number of OX-42–immunopositive cells 14 days after ischemia in the cerebral cortex. *N* = 12 per group. ^#^ *p* < 0.01 compared to the saline and control groups.

**FIGURE 6:**
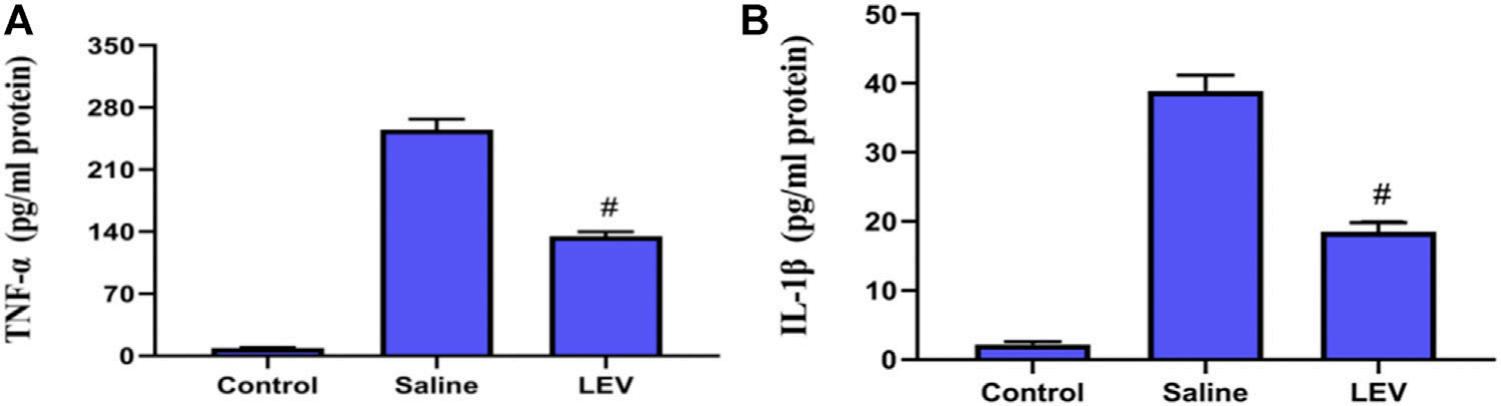
Levetiracetam (LEV) suppresses the production of IL-1β and TNF-α in the cerebral cortex of rats 14 days after ischemic brain injury. **(A**,**B)** Interleukin-1β (IL-1β) and tumor necrosis factor-α (TNF-α) concentrations in the cerebral cortex were measured by enzyme-linked immunosorbent assays (ELISA). *N* = 12 per group. ^#^ *p* < 0.01 compared to the saline and control groups.

OX-42 immunostaining of microglia showed that activated microglia appeared in the ipsilateral cortex after ischemia stroke (Figure 5A). After LEV treatment, the number of microglia with positive OX-42 expression was significantly reduced compared to the saline group (Figure 5B). To determine whether LEV suppressed the upregulation of pro-inflammatory factors induced by microglia activation, the levels of pro-inflammatory cytokines including IL-1β and TNF-α in the ipsilateral cortex were measured. Compared to the saline group, the levels of IL-1β and TNF-α in the LEV group were significantly reduced (Figures 6A,B).

### LEV Treatment Upregulated Pro-Angiogenic Factors in the Ischemic Brain and Increased the Level of HSP70

**FIGURE 7:**
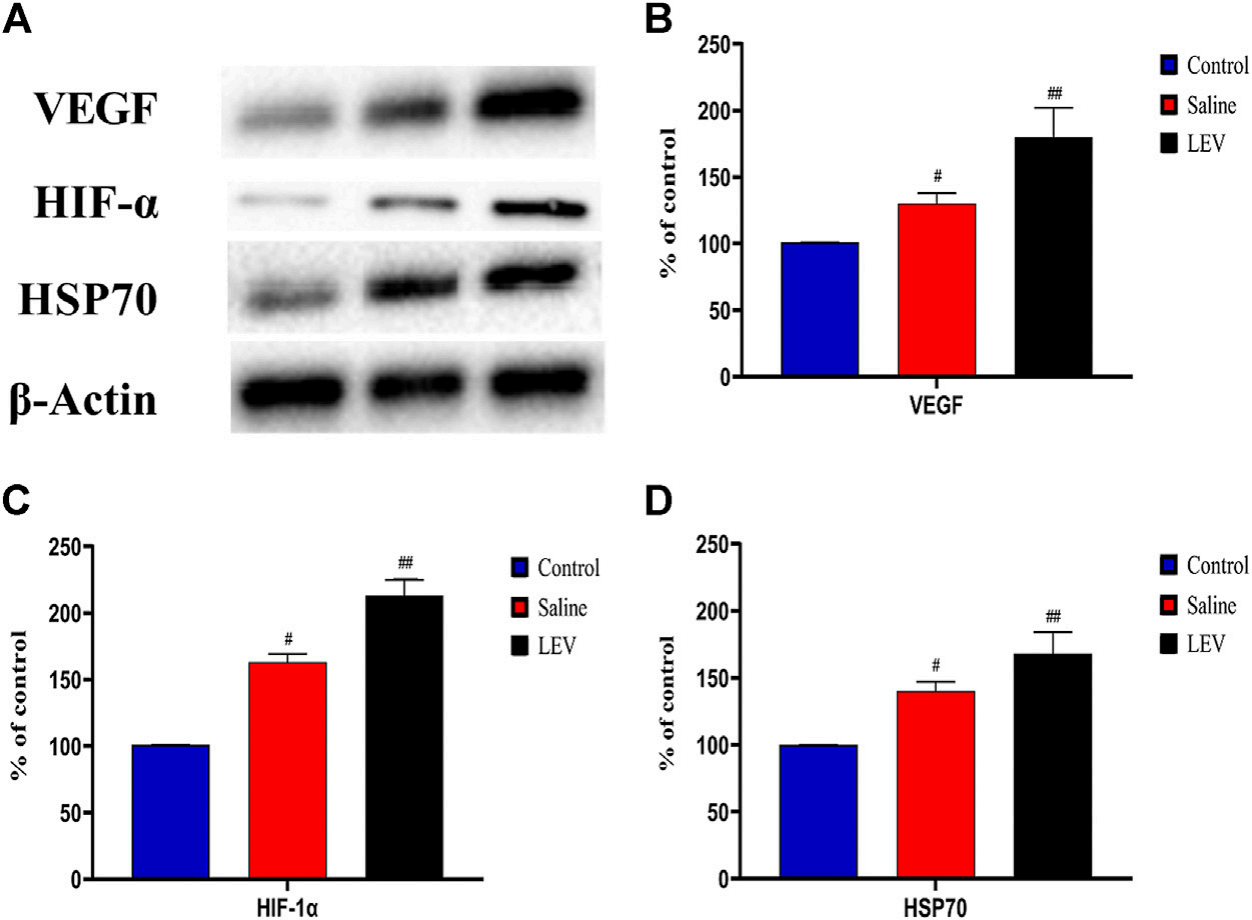
Levetiracetam (LEV) increased the levels of vascular endothelial growth factor (VEGF), hypoxia-inducible factor-1α (HIF-1α), and heat shock protein 70 (HSP70) in the cerebral cortex of middle cerebral artery occlusion (MCAO) rats on day 14. **(A)** The protein levels of VEGF, HIF-1 α, HSP70, and β-actin were measured by Western blot analysis. **(B,C)** Quantitative results of VEGF, HIF-1 α, and HSP70. *N* = 12 per group. ^#^ *p* < 0.01 compared to the control group; ^##^ *p* < 0.01 compared to the saline group.

To evaluate the role of HSP70 in the neuroprotective effect of LEV, we performed Western blotting of HSP70 in the ipsilateral cortex after ischemia stroke. Compared to the control group, the HSP70 level in the saline group increased, and this increase was enhanced after LEV treatment (Figures 7A,D). As expected, the HIF-1α and VEGF protein levels increased gradually in the MCAO-induced rats compared to the control group (Figures 7A,B,C). LEV therapy further enhanced this increase.

### Effect of LEV on Cell Toxicity Induced by OGD

**FIGURE 8:**
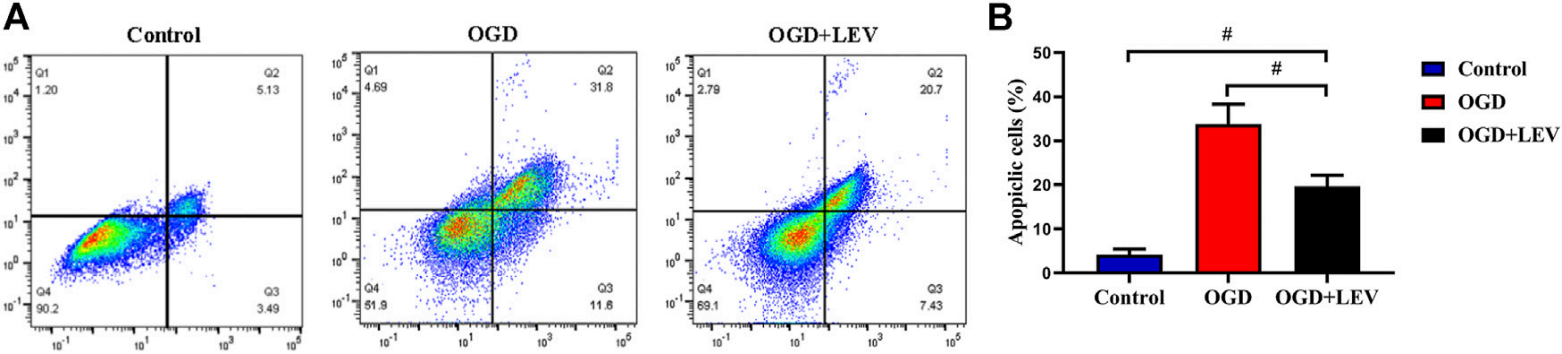
Quantitative analysis of neuronal cell apoptosis by flow cytometry. **(A)** The number of apoptotic cells in the oxygen-glucose deprivation (OGD) group was increased significantly compared to the control group, but levetiracetam (LEV) reduced the apoptosis induced by OGD. **(B)** Quantification of cell apoptosis in the different groups. ^#^ *p* < 0.01 compared to the saline and control groups.

OGD caused a significant increase in neuronal cell apoptosis when compared to the control group. In contrast, LEV preincubation inhibited OGD-mediated cell death (Figures 8A,B).

## Discussion

Accumulating evidence has established that ischemic stroke can lead to a dramatic loss of neurons. Cerebral cortex damage is accompanied by severe impairments in learning and memory functions (Carmichael et al., 2017; Faggi et al., 2018; Wang et al., 2018). Growing evidence has found that in animal models of ischemic stroke, some anticonvulsants reduced brain damage and improved functional outcomes (Bolland et al., 2018; Brookes et al., 2018; Shamsi Meymandi et al., 2018; Chen et al., 2019; Brigo et al., 2020). LEV may contribute to neuron protection after cerebral ischemia reperfusion. The possible mechanism may be related with perforin release (Zhang et al., 2016). LEV administration just after hypoxia-ischemia might possess some protective effects against myocardial damage and contractility (Gurgul et al., 2018). LEV injection suppresses nonconvulsive seizure occurrence for at least 24 h. This electrophysiological effect could explain the long-lasting reduction of ischemic brain damage caused by this drug (Cuomo et al., 2013). In this study, LEV significantly reduced the infarct volume and improved functional recovery in rats on the 14th day after MCAO. Notably, existing studies also suggest these results (Hanon and Klitgaard, 2001). At the same time, LEV also upregulated the protein levels of VEGF, HIF-1α, and HSP70 in the cerebral cortex; suppressed inflammation related to brain injury; and reduced neuronal apoptosis.

Studies have shown that angiogenesis after cerebral ischemia in humans and animals could help improve microcirculation in brain tissue (Hermann and Zechariah, 2009; Shimotake et al., 2010). In the early phase after stroke, LEV mitigates pathological endothelial cell function by increasing the permeability of the blood–brain barrier and promoting neurovascular reconstruction in the later recovery phase. Therefore, LEV has a dual role in preserving postischemic endothelial cell function after ischemia (Gurses et al., 2009; Itoh et al., 2016). In the current study, LEV treatment can significantly enhance postischemic angiogenesis by increasing microvessel density, suggesting that LEV could promote the formation and perfusion of microvessels (Lombardo et al., 2008). Our study showed that LEV treatment during the ischemic recovery period significantly enhanced the upregulation of VEGF, and the activity of VEGF protein levels was significantly increased. The results suggest that LEV enhanced the angiogenic ability after ischemia.

HIF-1α regulates pro-angiogenic genes after hypoxia-ischemia, and its activation is predominantly controlled by subunit stabilization (Ke and Costa, 2006). Our results showed that LEV treatment significantly enhanced MCAO-induced HIF-1α protein accumulation. These findings suggest that the angiogenic effect of LEV involved the regulation of HIF-1α, which is essential for the ability of LEV to improve functional recovery after ischemia.

It has become increasingly clear that the brain injury caused by an ischemic stroke is closely related to inflammatory responses, including the infiltration of mononuclear phagocytes (Kim et al., 2014). A previous study has shown that 3-day treatment with levetiracetam maintained SV2A protein expression *via* interaction with astrocytes, which influenced the OPC lineage through activation of CREB to protect white matter from ischemia. Microglia-mediated neuroinflammation plays an important role in ischemic brain injury (Mobarra et al., 2019). The overexpression of inflammatory cytokines (IL-1β and TNF-α), iNOS, and COX-2 induces the inflammatory response (Boehme et al., 2016; Couch et al., 2017; Piccardi et al., 2017). After a stroke, many microglia accumulate in the infarct area, leading to neuroinflammatory reactions (Xiong et al., 2016). Some studies have suggested that LEV may suppress the production of inflammatory cytokines by inhibiting NF-κB transcriptional activity (Mohammad et al., 2019).

Previous studies have shown that HSPs, including HSP70, were upregulated soon after global cerebral ischemia and persisted in the ischemic brain penumbra (Allende et al., 2016). In rat stroke animal models, the overexpression of HSP70 promoted the recovery of nervous system function after an ischemic stroke (Doeppner et al., 2017). Our results showed that LEV treatment caused a further increase in HSP 70 compared to the saline group. This was similar to several studies reporting that the level of HSP70 was significantly increased 1, 3, 24, and 72 h after ischemia (Wang et al., 2018). HSP70 may exert its antiapoptotic effects through a variety of mechanisms. It inhibits the activation of caspase-3 and its downstream factors, including phospholipase A2 (Pan et al., 2014; Wang et al., 2015; Bustamante et al., 2017). Therefore, the overexpression of HSP70 induced by LEV may play a key role in neuroprotection after an ischemic stroke.

In conclusion, our study demonstrated that LEV treatment significantly prevented neuronal cell death and promoted angiogenesis and the recovery of nervous system function after ischemia. The neuroprotection induced by LEV may involve a variety of mechanisms, including suppression of the inflammatory response induced by ischemia, increasing the expression of VEGF, HIF-1α, and HSP70, and reducing neuronal apoptosis.

## Data Availability

The raw data supporting the conclusion of this article will be made available by the authors, without undue reservation.

## References

[B1] Allende M. Molina E. Guruceaga E. Tamayo I. González-Porras J. R. Gonzalez-López T. J. (2016). Hsp70 Protects from Stroke in Atrial Fibrillation Patients by Preventing Thrombosis without Increased Bleeding Risk. Cardiovasc. Res. 110 (3), 309–318. 10.1093/cvr/cvw049 26976620

[B2] Anrather J. Iadecola C. (2016). Inflammation and Stroke: An Overview. Neurotherapeutics 13 (4), 661–670. 10.1007/s13311-016-0483-x 27730544 PMC5081118

[B3] Boehme A. K. McClure L. A. Zhang Y. Luna J. M. Del Brutto O. H. Benavente O. R. (2016). Inflammatory Markers and Outcomes after Lacunar Stroke. Stroke 47 (3), 659–667. 10.1161/strokeaha.115.012166 26888535 PMC4766076

[B4] Bolland M. J. Avenell A. Gamble G. Grey A. (2018). Reader Response: Expression of Concern: Does Compensatory Hyperparathyroidism Predispose to Ischemic Stroke? Decreased Bone Mass and Increased Bone Turnover with Valproate Therapy in Adults with Epilepsy; an Alternative to Vitamin D Supplementation to Prevent Fractures in Patients with MS; High Prevalence of Vitamin D Deficiency and Reduced Bone Mass in Parkinson's Disease. Neurology 90 (13), 627–628. 10.1212/wnl.0000000000005203 29581333

[B5] Brigo F. Lochner P. Nardone R. Manganotti P. Lattanzi S. (2020). Increased Risk of Stroke and Myocardial Infarction in Patients with Epilepsy: A Systematic Review of Population-Based Cohort Studies. Epilepsy Behav. 104 (Pt B), 106307. 10.1016/j.yebeh.2019.05.005 31182394

[B6] Brookes R. L. Crichton S. Wolfe C. D. A. Yi Q. Li L. Hankey G. J. (2018). Sodium Valproate, a Histone Deacetylase Inhibitor, Is Associated with Reduced Stroke Risk after Previous Ischemic Stroke or Transient Ischemic Attack. Stroke 49 (1), 54–61. 10.1161/strokeaha.117.016674 29247141 PMC5753817

[B7] Bustamante A. López-Cancio E. Pich S. Penalba A. Giralt D. García-Berrocoso T. (2017). Blood Biomarkers for the Early Diagnosis of Stroke. Stroke 48 (9), 2419–2425. 10.1161/strokeaha.117.017076 28716979

[B8] Carmichael S. T. Kathirvelu B. Schweppe C. A. Nie E. H. (2017). Molecular, Cellular and Functional Events in Axonal Sprouting after Stroke. Exp. Neurol. 287 (Pt 3), 384–394. 10.1016/j.expneurol.2016.02.007 26874223 PMC4980303

[B9] Chen P.-H. Tsai S.-Y. Pan C.-H. Chang C.-K. Su S.-S. Chen C.-C. (2019). Mood Stabilisers and Risk of Stroke in Bipolar Disorder. Br. J. Psychiatry 215 (1), 409–414. 10.1192/bjp.2018.203 30295208

[B10] Couch Y. Akbar N. Davis S. Fischer R. Dickens A. M. Neuhaus A. A. (2017). Inflammatory Stroke Extracellular Vesicles Induce Macrophage Activation. Stroke 48 (8), 2292–2296. 10.1161/strokeaha.117.017236 28536169 PMC5531255

[B11] Cuomo O. Rispoli V. Leo A. Politi G. B. Vinciguerra A. di Renzo G. (2013). The Antiepileptic Drug Levetiracetam Suppresses Non-convulsive Seizure Activity and Reduces Ischemic Brain Damage in Rats Subjected to Permanent Middle Cerebral Artery Occlusion. PloS One 8 (11), e80852. 10.1371/journal.pone.0080852 24236205 PMC3827478

[B13] Doeppner T. R. Doehring M. Kaltwasser B. Majid A. Lin F. Bähr M. (2017). Ischemic Post-Conditioning Induces Post-Stroke Neuroprotection via Hsp70-Mediated Proteasome Inhibition and Facilitates Neural Progenitor Cell Transplantation. Mol. Neurobiol. 54 (8), 6061–6073. 10.1007/s12035-016-0137-3 27699598

[B12] Dąbrowski J. Czajka A. Zielińska-Turek J. Jaroszyński J. Furtak-Niczyporuk M. Mela A. (2019). Brain Functional Reserve in the Context of Neuroplasticity after Stroke. Neural Plast. 2019, 9708905. 10.1155/2019/9708905 30936915 PMC6415310

[B14] Faggi L. Pignataro G. Parrella E. Porrini V. Vinciguerra A. Cepparulo P. (2018). Synergistic Association of Valproate and Resveratrol Reduces Brain Injury in Ischemic Stroke. Ijms 19 (1), 172. 10.3390/ijms19010172 PMC5796121 29316653

[B15] Grysiewicz R. A. Thomas K. Pandey D. K. (2008). Epidemiology of Ischemic and Hemorrhagic Stroke: Incidence, Prevalence, Mortality, and Risk Factors. Neurol. Clin. 26 (4), 871–895. 10.1016/j.ncl.2008.07.003 19026895

[B16] Gurgul S. Buyukakilli B. Komur M. Okuyaz C. Balli E. Ozcan T. (2018). Does Levetiracetam Administration Prevent Cardiac Damage in Adulthood Rats Following Neonatal Hypoxia/Ischemia-Induced Brain Injury?. Medicina 54 (2), 12. 10.3390/medicina54020012 PMC6037241 30344243

[B17] Gurses C. Ekizoglu O. Orhan N. Ustek D. Arican N. Ahishali B. (2009). Levetiracetam Decreases the Seizure Activity and Blood-Brain Barrier Permeability in Pentylenetetrazole-Kindled Rats with Cortical Dysplasia. Brain Res. 1281, 71–83. 10.1016/j.brainres.2009.05.033 19464270

[B18] Hanon E. Klitgaard H. (2001). Neuroprotective Properties of the Novel Antiepileptic Drug Levetiracetam in the Rat Middle Cerebral Artery Occlusion Model of Focal Cerebral Ischemia. Seizure 10 (4), 287–293. 10.1053/seiz.2000.0511 11466025

[B19] Hermann D. M. Zechariah A. (2009). Implications of Vascular Endothelial Growth Factor for Postischemic Neurovascular Remodeling. J. Cereb. Blood Flow Metab. 29 (10), 1620–1643. 10.1038/jcbfm.2009.100 19654590

[B20] Hong M. Shi H. Wang N. Tan H. Y. Wang Q. Feng Y. (2019). Dual Effects of Chinese Herbal Medicines on Angiogenesis in Cancer and Ischemic Stroke Treatments: Role of HIF-1 Network. Front. Pharmacol. 10, 696. 10.3389/fphar.2019.00696 31297056 PMC6606950

[B21] Inaba T. Miyamoto N. Hira K. Ueno Y. Yamashiro K. Watanabe M. (2019). Protective Role of Levetiracetam against Cognitive Impairment and Brain White Matter Damage in Mouse Prolonged Cerebral Hypoperfusion. Neuroscience 414, 255–264. 10.1016/j.neuroscience.2019.07.015 31302262

[B22] Itoh K. Ishihara Y. Komori R. Nochi H. Taniguchi R. Chiba Y. (2016). Levetiracetam Treatment Influences Blood-Brain Barrier Failure Associated with Angiogenesis and Inflammatory Responses in the Acute Phase of Epileptogenesis in Post-status Epilepticus Mice. Brain Res. 1652, 1–13. 10.1016/j.brainres.2016.09.038 27693413

[B23] Jayaraj R. L. Azimullah S. Beiram R. Jalal F. Y. Rosenberg G. A. (2019). Neuroinflammation: Friend and Foe for Ischemic Stroke. J. neuroinflammation 16 (1), 142. 10.1186/s12974-019-1516-2 31291966 PMC6617684

[B24] Kapur J. Elm J. Chamberlain J. M. Barsan W. Cloyd J. Lowenstein D. (2019). Randomized Trial of Three Anticonvulsant Medications for Status Epilepticus. N. Engl. J. Med. 381 (22), 2103–2113. 10.1056/nejmoa1905795 31774955 PMC7098487

[B25] Ke Q. Costa M. (2006). Hypoxia-inducible Factor-1 (HIF-1). Mol. Pharmacol. 70 (5), 1469–1480. 10.1124/mol.106.027029 16887934

[B26] Kim J. Y. Kawabori M. Yenari M. A. (2014). Innate Inflammatory Responses in Stroke: Mechanisms and Potential Therapeutic Targets. Cmc 21 (18), 2076–2097. 10.2174/0929867321666131228205146 PMC4104826 24372209

[B27] Lombardo L. Pellitteri R. Balazy M. Cardile V. (2008). Induction of Nuclear Receptors and Drug Resistance in the Brain Microvascular Endothelial Cells Treated with Antiepileptic Drugs. Cnr 5 (2), 82–92. 10.2174/156720208784310196 18473823

[B28] Mobarra N. Morovatdar N. Di Napoli M. Stranges S. Behrouz R. Amiri A. (2019). The Association between Inflammatory Markers in the Acute Phase of Stroke and Long-Term Stroke Outcomes: Evidence from a Population-Based Study of Stroke. Neuroepidemiology 53 (1-2), 20–26. 10.1159/000494685 30991382

[B29] Mohammad H. M. F. Sami M. M. Makary S. Toraih E. A. Mohamed A. O. El-Ghaiesh S. H. (2019). Neuroprotective Effect of Levetiracetam in Mouse Diabetic Retinopathy: Effect on Glucose Transporter-1 and GAP43 Expression. Life Sci. 232, 116588. 10.1016/j.lfs.2019.116588 31226418

[B30] Morris R. (1984). Developments of a Water-Maze Procedure for Studying Spatial Learning in the Rat. J. Neurosci. Methods 11 (1), 47–60. 10.1016/0165-0270(84)90007-4 6471907

[B31] Pan J. Liu H. Zhou J. Liu Z. Yang Y. Peng Y. (2014). Ipsilateral Hippocampal Proteomics Reveals Mitochondrial Antioxidative Stress Impairment in Cortical-Lesioned Chronic Mild Stressed Rats. Cmm 14 (9), 1186–1196. 10.2174/1566524014666141021143333 25336330

[B32] Piccardi B. Giralt D. Bustamante A. Llombart V. García-Berrocoso T. Inzitari D. (2017). Blood Markers of Inflammation and Endothelial Dysfunction in Cardioembolic Stroke: Systematic Review and Meta-Analysis. Biomarkers : Biochem. indicators Expo. response, susceptibility chemicals 22 (3-4), 200–209. 10.1080/1354750x.2017.1286689 28117601

[B33] Puig B. Brenna S. Magnus T. (2018). Molecular Communication of a Dying Neuron in Stroke. Ijms 19 (9), 2834. 10.3390/ijms19092834 PMC6164443 30235837

[B34] Ruan L. Wang B. ZhuGe Q. Jin K. (2015). Coupling of Neurogenesis and Angiogenesis after Ischemic Stroke. Brain Res. 1623, 166–173. 10.1016/j.brainres.2015.02.042 25736182 PMC4552615

[B35] Shamsi Meymandi M. Soltani Z. Sepehri G. Amiresmaili S. Farahani F. Moeini Aghtaei M. (2018). Effects of Pregabalin on Brain Edema, Neurologic and Histologic Outcomes in Experimental Traumatic Brain Injury. Brain Res. Bull. 140, 169–175. 10.1016/j.brainresbull.2018.05.001 29730418

[B36] Shao A. Zhou Y. Yao Y. Zhang W. Zhang J. Deng Y. (2019). The Role and Therapeutic Potential of Heat Shock Proteins in Haemorrhagic Stroke. J. Cel Mol Med 23 (9), 5846–5858. 10.1111/jcmm.14479 PMC6714234 31273911

[B37] Shetty A. K. (2013). Prospects of Levetiracetam as a Neuroprotective Drug against Status Epilepticus, Traumatic Brain Injury, and Stroke. Front. Neurol. 4, 172. 10.3389/fneur.2013.00172 24204362 PMC3816384

[B38] Shimotake J. Derugin N. Wendland M. Vexler Z. S. Ferriero D. M. (2010). Vascular Endothelial Growth Factor Receptor-2 Inhibition Promotes Cell Death and Limits Endothelial Cell Proliferation in a Neonatal Rodent Model of Stroke. Stroke 41 (2), 343–349. 10.1161/strokeaha.109.564229 20101028 PMC2846555

[B39] Stöllberger C. Finsterer J. (2016). Interactions between Non-vitamin K Oral Anticoagulants and Antiepileptic Drugs. Epilepsy Res. 126, 98–101. 10.1016/j.eplepsyres.2016.06.003 27450623

[B40] Tsai L.-K. Wang Z. Munasinghe J. Leng Y. Leeds P. Chuang D.-M. (2011). Mesenchymal Stem Cells Primed with Valproate and Lithium Robustly Migrate to Infarcted Regions and Facilitate Recovery in a Stroke Model. Stroke 42 (10), 2932–2939. 10.1161/strokeaha.110.612788 21836090 PMC3183311

[B44] Wang F. Tang H. Zhu J. Zhang J. H. (2018). Transplanting Mesenchymal Stem Cells for Treatment of Ischemic Stroke. Cel Transpl. 27 (12), 1825–1834. 10.1177/0963689718795424 PMC6300770 30251564

[B45] Wang T. Yu D.-R. Huang J. Liu Q. Wang D.-X. Luo N. (2018). Multimodal Rehabilitation Program Promotes Motor Function Recovery of Rats after Ischemic Stroke by Upregulating Expressions of GAP-43, SYN, HSP70, and C-MYC. J. Stroke Cerebrovasc. Dis. 27 (10), 2829–2839. 10.1016/j.jstrokecerebrovasdis.2018.06.018 30093210

[B43] Wang X. Luo Y. Sun H. Feng J. Ma S. Liu J. (2015). Dynamic Expression Changes of Bcl-2, Caspase-3 and Hsp70 in Middle Cerebral Artery Occlusion Rats. Brain Inj. 29 (1), 93–97. 10.3109/02699052.2014.945958 25158066

[B46] Wang Y. Wang B. Qi X. Zhang X. Ren K. (2019). Resveratrol Protects against Post-Contrast Acute Kidney Injury in Rabbits with Diabetic Nephropathy. Front. Pharmacol. 10, 833. 10.3389/fphar.2019.00833 31402864 PMC6675867

[B41] Wang Z. Leng Y. Tsai L.-K. Leeds P. Chuang D.-M. (2011). Valproic Acid Attenuates Blood-Brain Barrier Disruption in a Rat Model of Transient Focal Cerebral Ischemia: the Roles of HDAC and MMP-9 Inhibition. J. Cereb. Blood Flow Metab. 31 (1), 52–57. 10.1038/jcbfm.2010.195 20978517 PMC3049473

[B42] Wang Z. Tsai L.-K. Munasinghe J. Leng Y. Fessler E. B. Chibane F. (2012). Chronic Valproate Treatment Enhances Postischemic Angiogenesis and Promotes Functional Recovery in a Rat Model of Ischemic Stroke. Stroke 43 (9), 2430–2436. 10.1161/strokeaha.112.652545 22811460 PMC3429729

[B47] Warner J. J. Harrington R. A. Sacco R. L. Elkind M. S. V. (2019). Guidelines for the Early Management of Patients with Acute Ischemic Stroke: 2019 Update to the 2018 Guidelines for the Early Management of Acute Ischemic Stroke. Stroke 50 (12), 3331–3332. 10.1161/strokeaha.119.027708 31662117

[B48] Xiong X.-Y. Liu L. Yang Q.-W. (2016). Functions and Mechanisms of Microglia/macrophages in Neuroinflammation and Neurogenesis after Stroke. Prog. Neurobiol. 142, 23–44. 10.1016/j.pneurobio.2016.05.001 27166859

[B49] Xuan A. Long D. Li J. Ji W. Hong L. Zhang M. (2012). Neuroprotective Effects of Valproic Acid Following Transient Global Ischemia in Rats. Life Sci. 90 (11-12), 463–468. 10.1016/j.lfs.2012.01.001 22285595

[B50] Yang C. Hawkins K. E. Doré S. Candelario-Jalil E. (2019). Neuroinflammatory Mechanisms of Blood-Brain Barrier Damage in Ischemic Stroke. Am. J. Physiology-Cell Physiol. 316 (2), C135–C153. 10.1152/ajpcell.00136.2018 PMC6397344 30379577

[B51] Zhang Y. Li Y. Zuo L. Bao H. Xu X. Hao J. (2016). Levetiracetam Prevents Perforin Mediated Neuronal Injury Induced by Acute Cerebral Ischemia Reperfusion. Mol. Neurobiol. 53 (8), 5480–5491. 10.1007/s12035-015-9467-9 26454821

